# Measuring the impact of community-based interventions on type 2 diabetes control during the COVID-19 pandemic in Cape Town – A mixed methods study

**DOI:** 10.4102/safp.v64i1.5558

**Published:** 2022-08-18

**Authors:** Neal J. David, Graham Bresick, Natasha Moodaley, Klaus B. von Pressentin

**Affiliations:** 1 Division of Family Medicine, School of Public Health and Family Medicine, Faculty of Health Sciences, University of Cape Town, Cape Town, South Africa; 2 Metro District Health Services, Western Cape Department of Health, Cape Town, South Africa

**Keywords:** COVID-19, type 2 diabetes, home delivery of medication, glycaemic control, primary care, district healthcare, community health workers, mixed methods

## Abstract

**Background:**

The coronavirus disease 2019 (COVID-19) pandemic exposed the risks of poorly controlled noncommunicable diseases, especially in persons with diabetes. The pandemic outbreak in Cape Town, South Africa, required a rapid reorganisation of primary care services. Community-based measures were activated to ensure continuity of care by implementing home delivery of medication by community health workers. After five months of de-escalated chronic care, observations at an urban primary care facility suggested that noncommunicable disease patients had not overtly decompensated despite suspending regular in-facility services. This study attempted to understand what impact de-escalation of regular care and escalation of community-based interventions had on type 2 diabetes patients at this primary care facility.

**Methods:**

A mixed methods study design was used, consisting of data captured prospectively from diabetic patients who returned to the facility for routine care post-lockdown, as well as qualitative interviews to ascertain patients’ experiences of the home delivery service.

**Results:**

The data set included 331 (72%) patients in the home delivery group and 130 (28%) in the non-home delivery group. Regression analysis demonstrated a statistically significant relationship between home delivery and improved diabetic control (*p* < 0.01), although this may be because of confounding factors. The mean glycaemic control was suboptimal both at baseline and post-lockdown in both groups. Interviews with 83 study patients confirmed the acceptability of the home delivery intervention.

**Conclusion:**

The rapid reorganisation of primary care services illustrates the versatility of a functional community-oriented primary care service, although not fully developed yet, to adapt to emerging community healthcare needs in the pandemic era.

## Background

The coronavirus disease 2019 (COVID-19 or C-19) pandemic exposed the risks associated with poor noncommunicable disease (NCD) control, particularly amongst diabetics,^1,2^ amidst a global rise in NCD prevalence, including in South Africa (SA).^3^ Type 2 diabetes mellitus (T2D) and hypertension are the most common NCDs treated in primary care facilities (PCFs) in SA and frequently present as comorbidities. Approximately 82% of patients attending Cape Town PCFs have at least one chronic condition,^4^ limiting the capacity to provide care for other emergent conditions in overcrowded PCFs that are often understaffed.

The outbreak of C-19 in the Western Cape (WC) province in March 2020, therefore, required rapid reorganisation of PCF-based services to allow for physical distancing within facilities and to increase capacity for the anticipated influx of patients requiring acute care.^5^ Overcrowding led to a further concern that high-risk NCD patients, many of whom are elderly, would be exposed to C-19 when attending PCFs. Routine nonemergency PCF services in Cape Town, including scheduled chronic care visits, were therefore de-escalated, and community-based interventions were implemented.^5,6^ Primary care facilities headcounts were reduced and only some outpatient services were continued on a ‘see and treat’ (S&T) basis.

Home delivery of medication (HDM) by community health workers (CHWs) was implemented as a community-based measure to ensure ongoing NCD patient care.^7^ Prepackaged medication from the WC’s central Chronic Dispensing Unit (CDU) was delivered to patients’ homes without requiring the usual PCF-based clinical assessments. Community health workers screened patients for C-19 symptoms and other complaints in the community and referred them to PCFs only when necessary. These measures are aligned with the community-orientated primary care (COPC) approach that has been adopted by the WC’s Department of Health (WCDOH) as the cornerstone of a re-engineered primary health care (PHC) system.^8^

After five months of de-escalated NCD care (April–August 2020), shifts in service utilisation and clinical presentation patterns were reported by clinical team members based at one of the WCDOH PCFs, Hanover Park Community Health Centre (HPCHC). Overall, the emergency centre (EC) visits at HPCHC decreased by 36% over five months of lockdown in 2020, with similar reductions in trauma and nontrauma cases.^9^ Whilst the reduction in trauma cases could be ascribed directly to lockdown regulations such as a curfew and the ban on alcohol sales, the reasons for reduced nontrauma cases are less clear. These figures are aligned with other evidence from this period. A Cape Town district hospital study reported a 43% reduction in trauma cases and a 33% reduction in nontrauma cases over five weeks of lockdown compared to the same period pre-lockdown.^10^ The HPCHC team also noted that NCD patients had not overtly decompensated despite the suspension of regular in-facility services. It was also noted that fewer NCD-related emergencies presented to HPCHC in this period. The many potentially confounding factors notwithstanding, we hypothesised that some NCD patients may have benefited from the HDM service and that control of NCDs may have stabilised or even improved despite the suspension of facility-based care.

We sought to test this hypothesis by selecting a T2D study cohort as a proxy for the NCD population based on the following: (1) the high prevalence of T2D in the community, (2) the convenience of measuring T2D outcomes objectively with haemoglobin A1c (HbA1c) measurements and (3) the importance of glycaemic control in relation to C-19 risk. We aimed to determine the impact of de-escalated NCD care on T2D control in HPCHC patients by determining (1) if de-escalation was associated with any change in standard-of-care measures, including glycaemic control as reflected by HbA1c levels in T2D patients returning to routine PCF-based care; (2) whether HDM by CHWs improved disease control (as assessed by standard-of-care observations); and (3) whether patients receiving HDM (a) support this intervention and want it to continue, (b) feel that it improved their self-management during the intervention period and (c) supported the expansion of the service to include clinical monitoring and referral to PCFs as needed.

## Research methods and design

### Study design

We employed a two-phase mixed methods explanatory design.^11^ Quantitative data (standard-of-care observations) were first collected and analysed, followed by the collection and analysis of qualitative data to help explain or elaborate the quantitative results – the rationale being that quantitative data provide a general understanding of the problem, whereas qualitative data refine and explain statistically determined results by exploring participants’ views in more detail.

### Setting

Hanover Park Community Health Centre is one of the 51 PCFs in Cape Town. It provides comprehensive primary care to a community of approximately 50 000 people. Situated on the Cape Flats on the outskirts of Cape Town, the community is characterised by high levels of poverty, unemployment and gang-related crime. Before the pandemic, HPCHC had an average of 15 000 patient visits per month. Services include a routine outpatient department, clinics for NCDs, HIV, tuberculosis (TB) and mental health, an Emergencies Unit for medical emergencies and trauma, a Midwife Obstetric Unit (MOU) for antenatal, obstetrics and postnatal care, and allied medical services. In the pre-COVID era, the standard of care for stable NCD patients included repeat prescriptions for up to six months, coupled with clinical assessments by clinical nurse practitioners (CNPs) or medical officers (MOs), depending on the patient’s condition. Hanover Park Community Health Centre treats approximately 2600 diabetic patients annually, over 90% of whom have T2D.^12^

### Study population and sample

The study cohort comprised T2D patients returning to HPCHC for routine care from 01 September 2020 when lockdown restrictions were lifted. Hanover Park Community Health Centre pharmacy data showed that 71% of NCD patients had been issued with standard 6-month CDU prescriptions without clinical assessments during the lockdown period, and they were due to return to the clinic for assessment and repeat prescriptions within the study period.^13^ The balance of NCD patients was issued with 12-month CDU prescriptions based on a temporary departure from the legal maximum of six months for items not exceeding Schedule 5.^14^ Almost all T2D patients returning to HPCHC for routine NCD care during the study period were eligible. Eligibility criteria were as follows: an existing diagnosis of T2D and the inclusion of metformin and/or glimepiride on their CDU prescriptions during the lockdown period. These patients formed the quantitative data set.

With the start of the second C-19 wave in mid-December 2020, all non-emergency care (other than the S&T service) was again suspended. The study proceeded with the database that had been developed up to that point, and further study-related activities were completed remotely.

### Data collection

A standardised data collection sheet was used to capture standard-of-care observations in Microsoft Excel. Standard-of-care data and results of blood tests were extracted from the folders of eligible T2D patients returning to HPCHC between 01 September 2020 and 15 December 2020. Data included HbA1c levels on the day of the visit and from before the pandemic (i.e., pre-lockdown) to evaluate and compare glycaemic control before the first wave of C-19 and after the lockdown period. Consultation data on the day of return were also collected and captured. Data collection by HPCHC clinical staff members occurred during consultations (i.e., in real time) and was therefore dependent on operational factors. Additional data collected from pre- and post-lockdown visits included dates of visits, age, gender, weight, height (if available), blood pressure (BP) and pre-lockdown creatine levels to determine baseline kidney function (estimated glomerular filtration rate [eGFR]), HDM by a CHW during the lockdown, EC or hospital admissions during the lockdown period and confirmed (laboratory) diagnoses of COVID-19. Results of blood tests performed on the day of return were retrieved and included when they became available on the National Health Laboratory Service (NHLS) website.

A survey questionnaire was developed to determine patients’ experiences and preferences regarding HDM, their level of understanding regarding de-escalation of care, the impact of HDM and their preferences regarding future care options, including HDM. The questionnaire was developed by the principal investigator (PI), assisted by two not-for-profit organisation (NPO) nurse supervisors who coordinated the HDM service. The questionnaire, piloted with 10 HDM recipients, contained open-ended questions to allow for free-text responses and Likert scale questions. Pilot data were not included in our analysis. The questionnaire was subsequently refined by the study team to allow for optimal implementation. Every fifth patient in the quantitative data set was invited to complete the qualitative questionnaire if they met three additional criteria: (1) initial data extracted from the patient’s folder were complete, (2) a pre-lockdown HbA1c level had been taken within 12 months of the first lockdown and (3) the patient had received HDM during the lockdown period. Patients not meeting these criteria were excluded in favour of the next qualifying patient. On this basis, patients were selected for interview by the PI and research assistant (RA). Patient information leaflets and informed consent documents were available in the three main languages spoken in the WC. Consenting and interviews were initially conducted in person in a private room at the facility by the RA and subsequently telephonically because of the second lockdown.

### Data analysis

Quantitative data were periodically submitted to Percept Actuaries and Consultants for data analytic support. The data were exported to Stata 16 software,^15^ cleaned, analysed and shared with the study team in online meetings. Preliminary trend analysis was performed during the study by the data analysts together with the PI, and the final data set was reviewed by the whole study team. The hard copies of the data set (folder extracts) were numbered to correspond with the Excel spreadsheet and stored in a secure location. Following descriptive statistics to summarise the study sample data, we performed bivariate analysis to explore pairwise correlations between the variables of interest related to the study objectives (including comparing HDM and non-HDM groups), as well as multivariate analysis to analyse these relationships in more detail.

Qualitative interview responses were captured on RedCap and exported to Excel, cleaned and inductively coded manually and independently by two RAs with experience in qualitative data analysis.^16^ The data from the questionnaire were grouped to highlight the participants’ understanding of the de-escalation of routine services, the impact of the HDM service and their preferences.

### Ethical considerations

This study was approved by the University of Cape Town’s Human Research Ethics Committee (ref. no. 480/2020) and the Western Cape Provincial Health Research Committee to conduct the research onsite.

## Results

**FIGURE 1:**
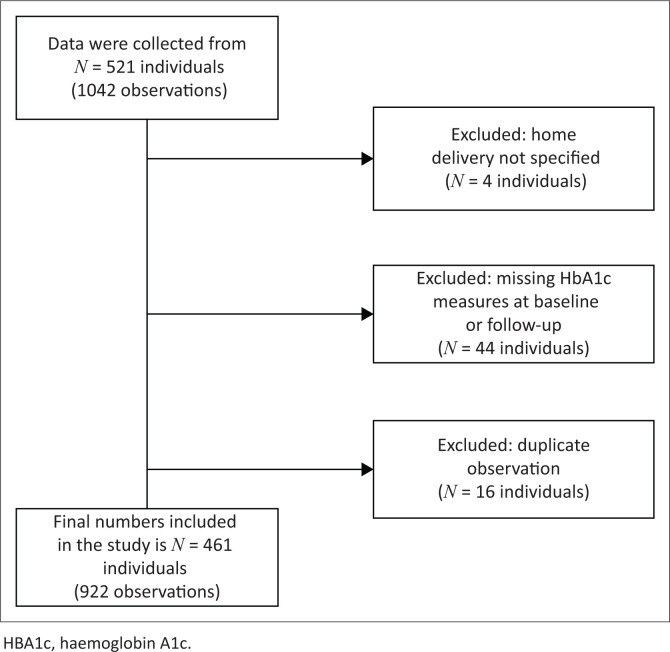
Flow diagram for exclusions from the data set.

Around 861 patients with T2D returned to HPCHC for routine care between 01 September and 15 December 2020 (between the first and second waves of C-19). Of this cohort, standardised data collection sheets were completed for 521 adult patients. When reviewing the standard-of-care data set for completeness, data for 64 patients had to be excluded (see Figure 1): 44 because of incomplete HbA1c measures, four where it could not be determined if HDM was received or not and 16 duplications. Of note is that four patients who were excluded had more than one exclusion factor. A total of 461 sets of patient data (922 observations) were eligible for analysis. As stated previously, it was not possible to enter more patients into the study after 15 December 2020 because of the second lockdown. The Human Research Ethics Committee (HREC) approved a protocol amendment to allow the remaining interviews to be performed telephonically with patients who had already been selected from the data set. The cohort of 461 eligible patients represents 54.1% of the T2D patients seen at the facility during the data capturing period and 17.5% of the total T2D patient population served by HPCHc in 2020.^17^ The average time difference between the baseline and follow-up HbA1c measures was 1.3 years (480 days) for the whole cohort (HDM and non-HDM patients). Eighty-three interviews with HDM patients (58 in-person and 25 telephonic) had been completed by the end of the data collection phase of the study, between 03 November 2020 and 23 March 2021.

The number of individuals excluded adds up to 64; however, one of these individuals had no home delivery status specified and had missing HbA1c measures, and three individuals had both missing HbA1c measures and were duplicate observations. Therefore, they had more than one exclusion criterion.

### Quantitative results of standard-of-care findings

#### Comparison of HDM and non-HDM groups at baseline and follow-up

Table 1 shows the summary statistics for the study population at baseline and follow-up. The HDM and non-HDM groups comprised 331 (72%) and 130 (28%) patients, respectively. HbA1c measures were lower in the HDM group at baseline and increased between baseline and follow-up in both groups. However, the increase in HbA1c was smaller in the HDM group. In both groups, the proportion classified as ‘well controlled’ (HbA1c of 7.5 or less) was lower at follow-up but fell by only 1% in the HDM group, compared to 5% in the non-HDM group.

**TABLE 1:** Summary statistics of the study population at baseline and follow-up.

Variables	Baseline								Follow-up							
	Non-HDM (*n* = 130)				HDM (*n* = 331)				Non-HDM (*n* = 130)				HDM (*n* = 331)			
	*n*	%	Mean	Range	*n*	%	Mean	Range	*n*	%	Mean	Range	*n*	%	Mean	Range
**Categorical variables**																
Female	100	77	-	-	254	77	-	-	100	77	-	-	254	77	-	-
Male	30	23	-	-	77	23	-	-	30	23	-	-	77	23	-	-
Well-controlled diabetics†	35	27	-	-	94	28	-	-	28	22	-	-	89	27	-	-
Non-well-controlled HbA1c	95	73	-	-	237	72	-	-	102	78	-	-	242	73	-	-
Clients with high BP‡	109	84	-	-	260	79	-	-	112	86	-	-	283	85	-	-
Clients without high BP‡	18	14	-	-	68	21	-	-	13	10	-	-	38	11	-	-
**Continuous variables**																
Age (years)	-	-	59	26–80	-	-	62	26–89	-	-	59	26–80	-	-	62	26–89
HbA1c	-	-	9.6	5.5–20	-	-	9.3	5.1–17.7	-	-	10.1	5.4–19.6	-	-	9.5	5.1–15.7
Weight (kg)	-	-	80	40–130	-	-	81	43–180	-	-	79	39–127	-	-	79	41–175

† , Well-controlled is classified as an HbA1c of 7.5 or less in this study;

‡ , The totals for baseline and follow-up blood pressure do not sum up to the sample totals because of missing values.

HDM, home delivery of medication; HbA1c, haemoglobin A1c; BP, blood pressure.

#### Correlations between the variables of interest

Table 2 shows the pairwise correlations between the variables of interest. HbA1c is seen to be inversely correlated with receiving home deliveries, meaning that HDM is associated with lower HbA1c measures. Age and weight also appear to be inversely related to HbA1c, which is unexpected given that diabetic control may be anticipated to be worse in the elderly and overweight or obese patients.

**TABLE 2:** Pairwise correlations matrix for variables of interest.

Variables	(1)	(2)	(3)	(4)	(5)	(6)	(7)
(1) HBA1c	1.000	-	-	-			-
(2) Received HDM (= 1)	−0.089*	1.000	-	-	-		-
(3) Follow-up (= 1)	0.065*	0.000	1.000	-	-		-
(4) Age	−0.186*	0.072*	0.000	1.000	-	-	-
(5) Female (= 1)	−0.029	−0.002	0.000	0.070*	1.000	-	-
(6) High BP (= 1)	0.080*	−0.050	0.103*	−0.003	−0.067*	1.000	-
(7) Weight	−0.061	0.004	−0.030	−0.169*	−0.166*	0.131*	1.000

* *p* < 0.1.

HBA1c, haemoglobin A1c; BP, blood pressure.

#### Multivariate analysis and comparisons between well-controlled and non-well-controlled groups

Multivariate analysis (Table 3) shows the results of three regressions analysing the relationship between HbA1c and HDM from baseline to follow-up. The first regression considers the whole study population, the second considers only the non-well-controlled diabetic group and the third considers only the well-controlled diabetic group. These three regressions include all available control variables: age, gender, BP and weight.

**TABLE 3:** Results of regression analysis.

Variables	Whole sample HBA1c	Non-well-controlled sample HBA1c	Well-controlled sample HBA1c
Received HDM (= 1)	−0.464** (−0.831 to −0.0977)	−0.380* (−0.732 to −0.0269)	−0.0646 (−0.272 to 0.143)
Follow-up (= 1)	0.266 (−0.0606 to 0.593)	0.330* (0.0116 to 0.648)	0.122 (−0.0496 to 0.294)
Age	0.186** (0.0755 to 0.296)	0.163** (0.0552 to 0.271)	0.0345 (−0.0291 to 0.0981)
Female (= 1)	−0.109 (−0.500 to 0.282)	−0.195 (−0.573 to 0.183)	0.308*** (0.0941 to 0.522)
High BP (= 1)	0.517* (0.0525 to 0.981)	0.0136 (−0.461 to 0.488)	0.0642 (−0.149 to 0.277)
Weight	−0.0157** (−0.0247 to −0.00664)	−0.0208** (−0.0295 to −0.0121)	0.0110** (0.00587 to 0.0161)

Note: Confidence intervals are reported in parentheses.

HBA1c, haemoglobin A1c; HDM, home delivery of medication.

* *p* < 0.05;

** *p* < 0.01.

For the whole study population, the HbA1c measures for the HDM group were 0.464% lower than in the non-HDM group, which is highly statistically significant (*p* < 0.01). Other statistically significant differences in this regression were age, high BP and weight. The relationship between HbA1c and age was nonlinear. As expected, high BP also had a positive relationship with HbA1c: clients with high BP on average had 0.517% higher HbA1c levels compared to those not classified as having high BP. Unexpectedly, weight had a statistically significant, inverse relationship with HbA1c, probably because of confounding factors that were not controlled for.

For the non-well-controlled diabetic group, all the relational effects were the same. The effects of HDM and age on HbA1c levels were still significant although at slightly lower levels. High BP was no longer significant in this regression.

In the well-controlled diabetic group, HDM did not appear to have a significant effect on HbA1c levels. The only variables that were significant in this (third) regression were gender and weight. Women had, on average, 0.308% higher HbA1c measures than men in the well-controlled sample. The relationship between weight and HbA1c is in the expected direction; a 1 kg increase in weight is associated with a 0.01% increase in HbA1c.

#### Summary of quantitative data findings

The mean HbA1c level for the whole T2D cohort reflected poor pre-lockdown diabetic control on average at 9.44%, with a 0.25% overall increment to 9.69% post-lockdown. This finding did not support our initial hypothesis. Notwithstanding, there are significant differences within the larger group that indicate different levels of control. The baseline pre-lockdown glycaemic control for the HDM cohort was better than that of the non-HDM cohort, with a mean HbA1c level of 9.3% versus 9.6%. This may reflect more favourable baseline conditions or a greater level of clinical stability even before HDM was implemented. However, the increment in the HbA1c level for the HDM group was 0.2% versus 0.5% for the non-HDM group. The disaggregation of the well-controlled patients (HbA1c < 7.5%) from the larger group shows that they comprised 28.0% of the HDM group pre-lockdown and 27.0% post-lockdown. The well-controlled proportion of the non-HDM group, on the other hand, was 27.0% pre-lockdown and 22.0% post-lockdown, representing a five times greater loss of well-controlled patients from the non-HDM group. In addition, although the overall incidence of laboratory-confirmed C-19 in this group of patients was small (10 patients, 2.2%), the incidence in the non-HDM group (four patients, 3.1%) was proportionately higher than that of the HDM group (six patients, 1.8%). This difference is not significant because of the small sample size. The headline findings (excluding C-19 incidence because of a lack of statistical significance) are summarised in Table 4.

**TABLE 4:** Summary of quantitative data findings.

Variables	Size of group		Pre-lockdown		Mean HbA1c		Post-lockdown		HbA1c		Difference in mean
	*n*	%	Mean	Range	*n*	%	Mean	Range	*n*	%	
Whole cohort	461	100	9.44	-	-	-	9.69	-	-	-	0.25 (increase)
HDM cohort	331	72	9.30	5.1–17.7	-	-	9.50	5.1–15.7	-	-	0.20 (increase)
Non-HDM cohort	130	28	9.60	5.5–20.0	-	-	10.1	5.4–19.6	-	-	0.50 (increase)
Well-controlled HDM cohort (HbA1c < 7.5%)	-	-	6.53	-	94	28	6.52	-	89	27	0.01 (decrease)
Well-controlled non-HDM cohort (HbA1c < 7.5%)	-	-	6.56	-	35	27	6.57	-	28	22	0.01 (increase)

HBA1c, haemoglobin A1c; HDM, home delivery of medication.

### Qualitative findings on the patient experience

The second study objective was to understand the experience of patients who were receiving HDM from the CHWs. The survey questionnaire was used to determine patient preferences with regard to receiving medication and chronic care – specifically where and how they would like to receive care in future. All participants (100%) expressed a clear preference for receiving HDM versus collecting medication at the clinic. Most patients (53%) cited convenience as the number one reason for wanting to receive medication by home delivery, followed by safety concerns, transportation challenges, and mobility (23%). Increased stress because of PCF overcrowding and long waiting times, as well as possible infection risks (12%), were also cited as reasons not to collect medication from the clinic. One patient cited difficulty accessing the clinic, noting not only the convenience of HDM but also how it saved on additional cost: ‘[*i*]t is very difficult for me to walk to the clinic – when I do go, I have to borrow a wheelchair from a neighbour for a fee’ (Patient 80, 81-year-old female, pensioner and HDM recipient).

When asked about alternative sites for the collection of medication within the community, such as a church hall or community centre, most patients (97%) were amenable to this with the proviso that the distance to the alternative site was closer to home than the clinic. Patient 25 (57-year-old female, unemployed and HDM recipient) responded: ‘I do not mind as long it is closer to my home and the lines are not long’, whereas it was more complicated for Patient 76, who stated that:

‘I will have to send my daughters to the civic [*community pre-packaged medication collection point*] to collect my medication, but they are not always available, so I really prefer home delivery.’ (Patient 76, 45-year-old male, employed full time and HDM recipient)

Whilst the convenience of having a collection site closer to home might mitigate challenges such as waiting times and the cost of transportation, it does not address the additional needs of physically challenged patients, or others who have conditions or responsibilities such as paid work that may need an after-hours service.

Most (94%) participants support receiving clinical care at home or at a support site within the community. When asked if they were able to manage their diabetes better if supported by CHWs who would refer them to the clinic, if necessary, 93% ‘agreed’ or ‘strongly agreed’ with this statement. However, 62% of this cohort showed a deterioration in their follow-up HbA1c levels. Some patients indicated that not having access to a glucometer was challenging and that the service could be improved should the CHWs have blood glucose tests available during their visits. Responses indicate that the service received from the CHW was generally better. Individualised care allowed them to spend more time with the CHW and ask questions that were related to other health challenges in the family, in addition to their chronic conditions. Participants also cited the experience as less stressful. Those who were at work and therefore did not interact with CHWs noted that the service was more convenient as they did not have to take a day off work just to collect medication. Overall, patients showed a strong preference for home delivery, as it mitigated some of the challenges that they faced when accessing their chronic medication in general and more specifically during the pandemic.

#### Summary of qualitative data findings

Based on the qualitative assessments of 83 HDM recipients from this database, it can be concluded that a CHW intervention is a highly acceptable and desirable healthcare component. The HDM service has fostered increased trust in the assistance that CHWs can provide. The headline findings from the interviews were as follows:

- All patients understood the need for clinic visits to be suspended because of C-19 and all felt that HDM worked well, primarily because they received reminders from CHWs to take their medication.
- Most patients felt that their blood glucose levels were better but did not attribute this to the HDM service directly.
- All patients wanted to continue with the HDM programme, either to save time and avoid exposure to the coronavirus at the clinic or because of safety concerns.
- Most patients indicated that the support from the CHW or nurse helped them to manage their diabetes better.

## Discussion

This study aimed to assess the impact of de-escalated in-facility services on patients with NCDs who received care at an urban PCF during the first lockdown period necessitated by the C-19 pandemic in 2020. Amongst the measures adopted to ensure uninterrupted NCD management, CHWs were deployed to deliver chronic medication to patients’ homes, enabling them to remain at home. When patients were permitted to return to the facility for review during a period of re-escalated in-facility care after the first wave had settled, standard-of-care folder extracts were obtained on 461 patients with T2D. This cohort of NCD patients was assessed to determine changes in disease control as well as any potential benefits that could be ascribed to the HDM service. Quantitative outcomes for the whole cohort were assessed and a qualitative survey review of patients receiving HDM was performed as part of a mixed methods study design. This study provided a unique opportunity to assess the status of patients who were temporarily denied access to their usual facility-based care but whose contact with CHWs was increased to maintain their stability and prevent clinical decompensation.

The quantitative analysis revealed that 72% of the cohort had received HDM during the lockdown period, whilst 28% had continued, for various reasons, to collect their medication from the facility. This proportion is aligned with the overall HDM project data in the greater Cape Town metropole, showing a 71.4% delivery rate in 42 participating facilities.^18^ The evidence in this cohort of returning T2D patients suggests that the strategy of de-escalated NCD care was largely successful, at least amongst those patients who received HDM. On average, HDM was positively associated with better glycaemic control when compared with patients not receiving HDM. The regression analysis demonstrated a statistically significant relationship between HDM and diabetic control (*p* < 0.01), although this may also be circumstantial and because of unintended selection bias at baseline. However, the greater retention of well-controlled patients in the HDM group is significant, as both groups had very similar proportions at baseline, with mean HbA1cs of 6.53% (HDM) versus 6.56% (non-HDM). Although there was a 0.03% difference, these cohorts were both optimally controlled for covariates, and it is, therefore, less likely that social or clinical circumstances impacting diabetic control would have applied to either group at baseline. In this sense, HDM was the most significant variable and was positively associated with good glycaemic control. Home delivery of medication may also have been protective against exposure to severe acute respiratory syndrome coronavirus 2 (SARS-CoV-2), as intended, with a higher incidence of C-19 in the non-HDM group (3.1% vs 1.8%), although the overall incidence was low in both groups, and the sample size was too small to determine any statistical significance.

It should be noted that baseline HbA1c levels for the whole cohort indicate poor glycaemic control, with a mean pre-lockdown level of 9.44% increasing to 9.69% post-lockdown. These levels are aligned with the long-term averages for the facility (based on a 10-year analysis) that show only 28.0% of patients have controlled levels of 6.0% – 8.0%, whilst 24.0% have levels of 8.0% – 10.0% and 41.0% have levels over 10.0%.^19^ This is broadly indicative of a system-wide and well-documented lack of effective control of NCDs such as T2D and hypertension at PCFs in Cape Town.^20^ These findings are consistent with other evidence throughout the country. In 2012, Amod reported that two-thirds of diabetic patients in South Africa had HbA1c levels above 7.5%,^21^ and in a recently published study, Piotie et al. showed that amongst diabetic patients on the chronic medication programme in Tshwane, only 29.2% achieved control, although this study used an HbA1c cut-off of 7.0% to determine control based on the 2017 Society for Endocrinology, Metabolism and Diabetes of South Africa (SEMDSA) guidelines.^22^

The qualitative assessments of patients receiving HDM showed a strong preference for the continuation of this service. There was also a broad expression of support for the clinical role that was played by the CHWs who provided the HDM service, with most patients describing their experience of HDM as better and less stressful than it would have been at the clinic. It is also interesting to note that many interviewed patients perceived their disease control to have improved during the lockdown period and felt that the support they received from the CHWs had helped them to manage their diabetes better, even though quantitatively 62% of these respondents had increased HbA1c levels post-lockdown. These patients were likely reflecting empowerment elements that they had developed in their interactions with the CHWs. Three patient empowerment domains are described in the literature: (1) patient activation, (2) patient self-management capacity and (3) psychosocial self-efficacy (also referred to as confidence).^23^ Empowerment is developed across these areas by elements of patient care that have been shown to be deficient or absent in PCFs, particularly in relation to NCDs.^24^ Based on the interview responses, it is highly likely that the three empowerment domains had improved amongst HDM recipients, although this was not necessarily associated with improved control. Sustained lower glycaemic levels will also require improvements in pharmacological management, particularly relating to the use of insulin.

The Diabetes Data Cascade for Hanover Park, prepared by the Provincial Health Data Centre, shows that only 35% of patients are provided with insulin and oral therapy, whilst 57% are on oral agents only.^12,17^ Patients on oral therapy are disadvantaged as they are not provided with glucometers and test strips, and very few can afford independent monitoring. As a result, most diabetic patients have no day-to-day knowledge of their glucose readings and are therefore less likely to pro-actively improve their glycaemic control. The problem is aggravated when primary care clinicians are reluctant to initiate insulin because of a lack of confidence in their own and their patient’s ability to manage insulin treatment.^25^ In addition, patients who are initiated on insulin according to PACK guidelines^26^ are prescribed dosages that are often subtherapeutic (e.g. < 0.1/kg per day), to titrate the dose upwards in conjunction with a self-monitoring diary. However, patients commonly fail to self-monitor effectively because of a lack of health education. Their prescriptions may then be repeated with insulin dosages that remain inadequate.

Notwithstanding the need to improve pharmacological management, the HDM initiative is well aligned with the commitment of the National Department of Health and the WCDOH to move towards a COPC approach to improve health services and save costs.^27^ Many initiatives have demonstrated the positive impact of CHW involvement in diabetic care as well as the cost-effectiveness of these interventions.^28^ However, there is little evidence available relating to the unique role that CHWs performed during a lockdown period – itself an unprecedented event. In the context described in this study, CHWs were required to ensure that patients were provided with their chronic medication by HDM and to act as the ‘eyes and ears’ of the health system by screening for any problems that might require escalation of care. In a healthcare service that remains predominantly facility-based and where implementation barriers have persisted since COPC was officially introduced in 2017 (in WC), the lockdown forced the use of a desired COPC model of service. In retrospect, the C-19 pandemic has allowed healthcare to leapfrog several obstacles in the COPC implementation process. To some extent, this in turn has made it possible for the researchers to assess the impact of the model during the lockdown period through the lens of the HDM service.

In the community context of the HPCHC service area, as in much of the Cape Flats, the adult prevalence of T2D has reached crisis proportions. Erasmus et al. found a prevalence of 28.2% in a comparable local population versus a pooled national prevalence of 15.25% in South African adults over 25 years of age.^29^ A combination of diabetogenic factors is evident, including poor dietary practices beginning in early childhood, often linked to socio-economics, and a food economy that promotes the consumption of cheap calories in the form of sugar and related processed food products rather than healthy alternatives. In addition, opportunities for physical exercise are restricted and the outdoor environment is frequently unsafe. Against this background, NCD patients are highly dependent on medication for control, and adherence is an important predictor of a positive long-term outcome.^30^ This study demonstrates that HDM, in at least ensuring a continuing supply of medication, may have resolved one potential barrier to adherence, that is, access to medication even when routine clinical services are de-escalated. It is equally important, however, that pharmacological management and patient empowerment elements are optimised to improve outcomes of NCD care and that of any other chronic condition. To this end, a novel approach to care may be possible by leveraging lessons learnt through our telemedicine experience during the pandemic.^31^ A project is currently being undertaken at HPCHC to provide remote support for poorly controlled diabetics via a telemedicine doctor and to link these patients with CHWs when required.^32^ Western Cape Government Health (WCDOH) is also piloting an e-locker system at several facilities for selected patients who are provided with a code via short message service (SMS) that will allow them to retrieve their medication at their convenience within a specified time period. Innovations of this nature are not only clinically indicated but are also critical to ensure that diabetic care is economically viable.

According to a 2019 cost-of-illness (COI) study, 49% of public sector spending on diabetes is related to managing complications,^33^ most of which are preventable by improving glycaemic control. Compounding this problem is the small proportion of T2D patients in South Africa who are diagnosed and treated. In the public sector, which provides care to 85% of the population, there are a mere 240 000 diabetic patients in care, representing only 5.2% of diabetic cases in SA, and 60.0% – 70.0% of these patients are not controlled. It is therefore likely that the direct costs of treating diabetes complications are significantly higher as a result of emergency care provided to undiagnosed patients.^34^ The authors of the COI study conclude that effective implementation of primary prevention, as well as secondary prevention in the form of targeted screening and improved pharmacological management of T2D, is urgent and must be prioritised. The valuable role of CHWs in the HDM service is indicative of the potential to achieve this goal by leveraging the advances that have been made to improve outcomes for patients receiving chronic disease management. The critical need to improve clinical outcomes in T2D is highlighted by the increasing prevalence of micro- and macrovascular disease complications and the well-established link between these and poor glycaemic control.^35^

Daviaud et al. have conservatively estimated that a CHW intervention in diabetic screening and management will increase the diagnosis rate by 7.0% and control by 7.0%, which would add 6.9 years of life expectancy and avert almost 1.2 million disability-adjusted life years (DALYs) over 10 years.^28^ Based on the current CHW remuneration of R3500 per month, this could be achieved at a cost for every DALY averted of R6096. The World Health Organization considers an averted DALY cost-effective at any point below per capita gross domestic product (GDP). At 8.0% of GDP; this intervention would not only be highly cost-effective but would also remain so at a far higher CHW salary level. In combination with the WCDOH’s existing community-based wellness initiative known as WoW! (Western Cape on Wellness), which has demonstrated a significant impact amongst participants,^36^ a CHW intervention would be well-placed to achieve the essential change in the NCD landscape that is so urgently required.

The results of this study suggest that there is a unique opportunity available in the aftermath of the pandemic to effectively improve diabetic control (and potentially that of other chronic conditions) by harnessing the advantages that have been demonstrated by the HDM service and combining these with improved pharmacological management, particularly relating to the introduction and titration of insulin. Not only would this provide a natural bridge between facility-based and community-based services, but it would also open the door to other innovative strategies, such as the telemedicine initiatives, that have emerged during the pandemic.

### Limitations

This study was limited by several factors, including the difficulty of performing in-person research during the pandemic. This was only possible in the brief window period between the end of the first wave and the start of the second wave, when the clinic closed for nonemergency care for the second time. Data collection was prematurely discontinued; only the data set available at that point could be used for analysis, and a switch to telephonic interviews was required for the remaining qualifying patients. Selection bias may have played a role in the finding that HDM was positively associated with improved glycaemic control because the baseline (pre-lockdown) levels of the HDM group are slightly better than the non-HDM group (9.3% vs 9.6%). This may reflect factors that were not assessed in this study, such as differing socio-economic circumstances. This is an initial exploratory study at a single facility and is limited by potential confounding factors. As such, the results may not represent the regional or national situation. A follow-up study using a more detailed questionnaire has been carried out in four PCFs in the Cape Town metropolitan area. This will determine a wider range of indicators and experiences of NCD patients during the lockdown periods and may address limitations identified in this study.

## Conclusion

The emergence of the COVID-19 pandemic required a rapid response to the challenge of managing patients with pre-existing chronic conditions. This included de-escalating routine PCF-based NCD care to community-based care to reduce the risk of exposure to C-19. The results of this study show a statistically significant positive association between glycaemic control and a HDM intervention in T2D patients returning to the facility after the first lockdown. The intervention was highly valued by participants. Their responses also reflect elements of patient empowerment and self-management capacity linked to the supportive role played by CHWs in this period. Poor baseline levels of glycaemic control suggest serious system-wide challenges requiring innovative disease-management strategies to improve outcomes. Further research is required to identify such strategies and services.
